# From Coordination to Personalization: A Trust-Aware Simulation Framework for AI-Driven Personalized Decision Support in Emergency Departments

**DOI:** 10.3390/jpm15120574

**Published:** 2025-11-28

**Authors:** Zoi Lygizou, Dimitris Kalles

**Affiliations:** School of Science & Technology, Hellenic Open University, 26 335 Patra, Greece

**Keywords:** personalized medicine, AI-driven decision support, simulation-based decision support, computational trust models, task allocation, emergency department (ED), healthcare staffing and coordination, multi-agent systems, workflow optimization, patient safety, resource utilization

## Abstract

**Background/Objectives**: Efficient and personalized task allocation in hospital emergency departments (EDs) is critical for operational efficiency and patient-centered care. However, the complexity of staff coordination and the variability among patients and healthcare professionals pose significant challenges. This study proposes a simulation-based framework for modeling doctors and nurses as intelligent agents guided by computational trust mechanisms. The objective is to explore how trust-informed coordination can support AI-driven and personalized decision-making in ED management. **Methods**: The framework was implemented in Unity, a 3D graphics platform, where agents assess their competence and patient-specific needs before undertaking tasks and adaptively coordinate with colleagues. The simulation environment enables real-time observation of workflow dynamics, resource utilization, and patient outcomes. We examined three scenarios—Baseline, Replacement, and Training—reflecting alternative staff management strategies. **Results**: Trust-informed task allocation balanced patient safety and efficiency by adaptively responding to nurse performance and patient acuity levels. In the Baseline scenario, prioritizing safety reduced errors but increased patient delays compared to a FIFO policy. The Replacement scenario improved throughput and reduced delays, though at additional staffing costs. The training scenario fostered long-term skill development among low-performing nurses, despite short-term delays and risks, supporting sustainable and personalized capacity building in ED teams. **Conclusions**: The proposed framework demonstrates the potential of computational trust for personalized and evidence-based decision support in emergency medicine. By linking staff coordination with adaptive and AI-informed decision-making, hospital managers are provided with a tool to evaluate alternative staffing and treatment policies under controlled and repeatable conditions. This work thus contributes to the broader vision of precision and personalized medicine, where operational decisions dynamically adapt to both patient needs and staff capabilities.

## 1. Introduction

Emergency Departments (EDs) are among the most overcrowded units in modern healthcare systems, and overcrowding has been recognized as a global challenge for more than two decades [1,2]. Patients arrive with highly diverse medical conditions and varying levels of acuity, yet EDs are obligated to provide care at the highest possible standard for all. Errors in ED procedures can have severe consequences, including disability or death. At the same time, the majority of patients—particularly within public EDs –face long lengths of stay, while hospital managers struggle with persistent budgetary and staffing constraints [3,4,5,6].

Public hospitals often operate under multiple, and sometimes conflicting, constraints such as limited budgets, shortages of skilled personnel, and restricted access to medical equipment. These challenges make it difficult to ensure both patient safety and operational efficiency. Because EDs operate continuously, including holidays, it is practically impossible to interrupt or reconfigure real ED workflows for the sole purpose of evaluating performance and efficiency [7,8,9]. Recent studies have explored partial flexibility in staffing, where nurses or servers can be reassigned at discrete intervals, such as the beginning of shifts, to better match demand while respecting operational constraints [10,11]. Additionally, simulation and machine learning—based approaches have been shown to optimize resource scheduling, reduce patient length of stay, and improve overall operational efficiency in EDs [12,13]. As a result, hospital managers require robust decision-support tools that enable evidence-based evaluation of alternative policies and interventions. Such tools should enable managers to systematically explore trade-offs between patient safety, operational efficiency, and staff development under controlled, repeatable conditions—ultimately allowing them test potential solutions before implementing changes in real ED environments [3,4,14,15].

Beyond improving operational efficiency, such decision-support frameworks are directly relevant to the goals of personalized medicine [16]. In the emergency department setting, personalization extends not only to diagnosis and treatment, but also to the allocation of healthcare resources in real time. Recent work has shown how machine learning can be used to personalize patient flow pathways and improve throughput in the ED [10]. Each patient arrives with unique clinical needs and acuity levels, and delays in care—caused, amongst a variety of reasons, by suboptimal task allocation or staffing mismatches—translate into heterogeneous risks at the individual level. By integrating computational trust models with AI-driven predictive techniques, it becomes possible to dynamically adapt the distribution of staff and tasks to match the evolving needs of specific patients and clinical contexts—creating a foundation for personalized decision support in emergency care. In this way, system-level optimization contributes directly to patient-specific outcomes, aligning workflow efficiency with the principles of personalized medicine. Future extensions of our framework could further strengthen this alignment by incorporating patient-level data (e.g., triage information, medical histories, or real-time vital signs) into the trust-based allocation process, enabling both personalized staffing strategies and individualized clinical care.

In dynamic work environments such as a hospital emergency department, the problem of task allocation closely resembles challenges observed in open multi-agent systems (MASs), where agents must self-organize under uncertainty and resource constraints. Modern computational trust and reputation models have been successfully applied to such problems in diverse domains, including peer-to-peer networks, online marketplaces, and the Internet of Things. In our previous work, we introduced the biologically inspired *Create Assemblies* (CA) trust model, which draws inspiration from synaptic plasticity in the human brain [17]. Unlike traditional models where the trustor (service requester) selects a trustee (service provider), CA enables the trustee to autonomously assess whether it possesses the necessary skills to undertake a task. More specifically, task requests are broadcast to potential trustees, which locally store the request and update their trust weights based on performance feedback after task completion. This event-driven mechanism supports adaptive task allocation while preserving efficiency and robustness. Subsequently, we improved CA by incorporating self-assessment: after providing a service, a trustee re-evaluates its performance, and if it falls below a predefined threshold, it (auto-)classifies itself as an unreliable provider [18]. This modification ensures that each provider maintains an up-to-date evaluation of its own capabilities, enabling the immediate detection of performance drops that could be harmful.

Motivated by these foundations, in this work we proposed a simulation-based framework that leverages computational trust models to support decision-making in emergency department staffing and workflow coordination. The framework, implemented in Unity, enables the modeling of doctors and nurses as intelligent agents operating under high variability and uncertainty, whose interactions are guided by trust-based task allocation mechanisms. Unlike traditional rule-based simulations, our approach focuses on capturing the dynamics of interpersonal trust and its impact on team performance, patient safety, and resource utilization.

By embedding trust models into agent decision-making, the system allows us to explore how different staffing configurations and training interventions affect both operational efficiency and patient-centered outcomes. In particular, the framework supports the evaluation of alternative policies under controlled and repeatable conditions, thereby offering hospital managers a tool for evidence-based experimentation without disrupting real-world clinical operations.

A key contribution of this work lies in bridging operational coordination with the principles of personalized and precision medicine. We demonstrate how adaptive task allocation, informed by computational trust and guided by AI reasoning, can evolve toward patient-specific and context-aware decision-making by dynamically aligning staff expertise and reliability with individual patient needs. This positions our simulation not only as a tool for operational research, but also as a stepping stone toward AI-driven, personalized decision-support in emergency medicine. Overall, the proposed framework advances the integration of artificial intelligence, simulation, and computational trust into the domain of personalized healthcare operations, illustrating how data-driven coordination can enhance both patient-specific outcomes and systemic efficiency.

In this work, we further clarify the distinction between operational personalization and clinical personalized medicine. The former focuses on the adaptive coordination of hospital resources, staff assignments, and workflows based on contextual data and performance data, whereas the latter targets individualized diagnosis and treatment guided by patient-specific biological and clinical information. By articulating this distinction, our framework positions operational personalization as an enabling layer that operationalizes the principles of clinical personalized medicine within healthcare delivery systems.

The remainder of this paper is organized as follows: Section 2 reviews related work across four key areas: simulation of EDs, decision-support systems in healthcare, computational trust models, and personalized medicine with adaptive staffing. Together, these perspectives provide the context and motivation for the proposed Unity-based simulation framework which is presented in Section 3. Section 4 introduces a case study with experiments comparing baseline, replacement, and training scenarios. Section 5 discusses results and limitations, while Section 6 concludes and outlines future research directions.

## 2. Related Work

Research on emergency department operations spans multiple methodological traditions, ranging from simulation-based and AI-enhanced approaches to decision-support systems and adaptive frameworks inspired by personalized and precision medicine. Simulation studies have modeled patient flows, staffing policies, and resource constraints, while decision-support systems increasingly incorporate machine learning and optimization to enhance real-time management. At the same time, computational trust models –though widely applied in multi-agent systems—have only recently begun to appear in healthcare research, leaving intra-organizational collaboration and task allocation largely unexplored. Finally, emerging work on personalized medicine highlights the need to align operational decision-making with patient-specific characteristics. In this section, we review four key areas of related work: simulation of emergency departments, decision-support systems in healthcare, computational trust models, and personalized medicine and adaptive staffing. Together, these perspectives provide the foundation for our proposed framework, while also revealing critical gaps at the intersection of trust, adaptive decision-making, and patient-centered resource allocation.

### 2.1. Simulation of Emergency Departments

Simulation is a widely used tool for analyzing Emergency Department (ED) operations, including patient flow, resource allocation, and bottleneck identification. The main approaches are Discrete-Event Simulation (DES), Agent-Based Simulation (ABS), and System Dynamics (SD), each with distinct strengths and limitations.

DES models patient arrivals, service times, and resource constraints, proving effective for evaluating interventions to reduce waiting times and overcrowding. For instance, Hamza et al. [3] proposed SIM-PFED, a hybrid DES-ABS model integrated with a multi-criteria decision-making approach (TOPSIS) to optimize patient flow and reduce throughput time in EDs. Kim [12] integrated DES with machine learning for dynamic physician allocation, while Castanheira-Pinto et al. [13] combined DES with lean healthcare principles to identify ED bottlenecks. Other works, such as De Santis et al. [5], have focused on DES calibration, and Dosi et al. [6] demonstrated how DES can be integrated with design thinking to guide organizational changes in ED operations. Collectively, these studies highlight DES as a robust and flexible tool for modeling ED processes, though it often abstracts away the nuances of individual staff behaviors and interpersonal interactions.

ABS extends traditional simulation by modeling patients, doctors, and nurses as autonomous agents with individual attributes and decision-making rules. This enables exploration of heterogeneous behaviors and interactions. Recent studies illustrate ABS for Digital Twin frameworks [4], modular, reusable environments [15], and stakeholder-friendly modeling languages [7]. ABS has been applied to study spatial layout effects on nursing efficiency [8,9] and team cognition [19], capturing contextual and human-centered factors. Yet, such perspectives remain underrepresented in ED-focused simulation research, where operational models rarely capture the underlying cognitive and relational processes that drive team performance.

SD approaches provide a high-level perspective, capturing the feedback loops and accumulations inherent in ED operations. SD models have been used for patient inflow-outflow dynamics [20], interventions for older patients [21], workplace violence and burnout [2], and participatory approaches in resource-limited settings [14]. Although SD can reveal structural issues affecting system performance, it generally lacks the granularity needed to capture individual-level variability or personalized interventions.

Overall, the literature demonstrates that simulation is a well-established and valuable tool for improving ED efficiency and patient flow. Yet, most existing work focuses on operational metrics and structural optimization, while interpersonal and team dynamics, as well as patient-specific decision-making, remain underexplored. Our work addresses this gap by incorporating computational trust models and agent-specific behaviors, providing a simulation framework that supports not only operational efficiency but also AI-driven, personalized, and adaptive staffing strategies in line with emerging paradigms in precision medicine and intelligent healthcare operations.

### 2.2. Decision Support Systems in Healthcare

Hospital managers increasingly rely on Decision Support Systems (DSS) to plan resources, schedule staff, and optimize patient flow. Approaches range from queuing theory and mathematical optimization to AI-driven scheduling algorithms.

For example, Yousefi and Yousefi [1] proposed a metamodel-based simulation optimization framework for staffing allocation in a Brazilian ED, combining an ensemble metamodel—integrating Adaptive Neuro-Fuzzy Inference System (ANFIS), Feed Forward Neural Network (FNN), and Recurrent Neural Network (RNN) with Adaptive Boosting (AdaBoost)—with a discrete Imperialist Competitive Algorithm (ICA), achieving a 24.82% reduction in door-to-doctor time. Similarly, Kim [12] integrated DES with machine learning to dynamically allocate physicians, reducing patient length of stay, while Ortiz-Barrios et al. [22] combined Extreme Gradient Boosting (XGBoost) with DES to predict treatment probabilities and adapt nurse staffing in Spanish EDs, reducing waiting times by up to 7.5 h. Castanheira-Pinto et al. [13] combined DES with lean healthcare principles to optimize staffing and process design in a public hospital ED, and De Santis et al. [5] improved timestamp data quality through simulation-based optimization using Weilbull distributions and derivative-free methods. Wang et al. [23] introduced a two-stage simulation-based optimization framework incorporating part-time shifts and nonlinear stochastic programming, showing that adaptive scheduling during peak hours reduces patient waiting times and resource strain. Similarly, Redondo et al. [24] addressed long-term capacity planning under demand uncertainty through Sample Average Approximation, supporting robust staffing alignment over extended horizons. Beyond purely algorithmic interventions, Dosi et al. [6] demonstrated how combining design thinking with DES can guide participatory organizational changes in EDs, enabling measurable improvements in patient waiting times and staff work quality within 18 months.

Together, these studies illustrate the potential of simulation—and AI—based DSS to enhance operational efficiency, optimize staffing and scheduling decisions, and support real-time, data-driven management in complex ED environments. However, most existing DSS frameworks do not fully account for uncertainty in staff behavior, variations in skill levels, or interpersonal trust relationships, which can critically affect performance in real ED settings.

Recent studies are also beginning to explore how DSS frameworks can move beyond static optimization to support personalized operational decisions, where predictive models dynamically adapt to patient-specific risks, acuity levels, and resource demands. However, such AI-based personalization remains largely reactive—adapting to outcomes after they occur—rather than proactive, where decisions anticipate performance variability before task execution. Our simulation-based, trust-aware framework addresses this gap by using continuously updated trust and competence profiles to predict task success likelihoods in advance, enabling anticipatory and personalized allocation of staff resources.

### 2.3. Computational Trust Models

Computational trust models, originate from computer science and multi-agent systems, with applications in e-commerce, collaborative robotics, and distributed decision-making. These models formalize trust relationships between agents to support more reliable coordination under uncertainty. Despite their widespread use in other domains, applications of computational trust in healthcare remain rare. Recent work by Anjana and Singhal [25] begins to address this gap by simulating trust dynamics in healthcare networks through a multi-agent, game-theoretic approach. Their study models interactions among hospitals, doctors, researchers, insurance companies, and patients in an Electronic Medical Record (EMR)-sharing environment, demonstrating how trust relationships evolve toward equilibrium through repeated cooperation and defection strategies. While this work highlights the relevance of computational trust for policy-making and system design in healthcare ecosystems, it primarily focuses on inter-organizational trust management. By contrast, intra-organizational dynamics such as staff collaboration and task allocation in EDs remain largely unexplored, leaning a significant gap in simulation-based decision-support research. Integrating computational trust with AI-driven personalization could enable systems that adapt coordination policies to the evolving needs of both staff and patients—a direction that motivates the present study.

### 2.4. Personalized Medicine and Adaptive Staffing

Personalized medicine traditionally focuses on tailoring diagnosis and treatment to individual patient characteristics. With the advent of artificial intelligence and data-driven modeling, the concept has expanded toward operational personalization, where resource allocation and workflow decisions adapt to each patient’s context. Savchenko and Bunimovich-Mendrazitsky [26] investigate the economic feasibility of personalized medicine for healthcare service providers, using bladder cancer as a case study, and propose a framework for identifying patient cohorts to balance clinical effectiveness with operational efficiency. Building on this idea, Gummadi [16] proposes a multimodal AI framework that integrates Electronic Health Records, patient-reported outcomes, genomic data, and real-time physiological information from wearable sensors to create comprehensive patient profiles. This approach not only supports personalized treatment but also informs workflow optimization and staffing decisions, demonstrating improvements in clinical outcomes, patient satisfaction, and provider efficiency.

In the context of emergency care, Hodgson et al. [10] applied AI and machine learning to optimize ED patient flow through a personalized vertical processing pathway, showing how individualized risk scores can support flexible, patient-specific care protocols that enhance throughput without compromising safety. Complementing such AI-driven interventions, Chan et al. [11] study dynamic server assignment in multiclass queueing systems with shift-based reassignment constraints, motivated by nurse staffing in EDs. Their analysis demonstrates that partial flexibility—where reassignment is possible only at shift boundaries—can substantially reduce waiting costs compared to static staffing policies, while remaining operational feasible. Together, these studies demonstrate that personalized, patient-centered approaches can be extended beyond clinical interventions to influence operational planning, resource allocation, and adaptive staffing in complex healthcare environments. In practice, adaptive staffing operates not only at the strategic scheduling level but also within shifts, where real-time coordination allows teams to reallocate tasks in response to changing performance or workload conditions. Our framework focuses on this intra-shift adaptability, modeling how trust-informed task redistribution can proactively maintain efficiency and safety without requiring large-scale rescheduling.

Despite these advances, most existing frameworks focus on predictive or reactive decision-making, leaving a gap for simulation-based approaches that combine personalized patient data with computational trust and adaptive staffing models to optimize both efficiency and team performance in real-time. This study addresses these gaps by introducing a Unity-based simulation framework that incorporates computational trust models to explore their potential impact on ED efficiency, staff development, and patient-centered outcomes—thereby contributing to the broader vision of AI-supported personalized healthcare operations.

## 3. Materials and Methods

### 3.1. Simulation Framework in Unity

#### 3.1.1. Overview

To systematically study and evaluate different, potentially personalized decision-making strategies and behavioral patterns in a clinical context, we developed a simulation framework using the Unity game engine. The environment models the daily workflow of hospital staff, focusing particularly on the interactions between doctors, nurses, and patients within an emergency room (ER) setting. The Unity-based implementation enables the execution of realistic, real-time agent behaviors and facilitates the comparison of alternative policy scenarios—including adaptive and personalized decision-support strategies—in a controlled and repeatable manner.

#### 3.1.2. Agents and Roles

The simulation comprises four primary agent categories:

- Doctors—evaluate patients and generate task requests (e.g., inserting an IV catheter), according to their assigned evaluation style. Each task request includes an estimated difficulty level and expected execution duration, derived from the doctor’s assessment of the patient’s condition;
- Nurses—receive and prioritize task requests based on a configurable decision policy (e.g., CA trust model, First-In-First-Out (FIFO)). Nurses then evaluate whether to accept or ignore each request. Their performance in executing accepted tasks is affected by their skill level (nurse quality) and in, certain scenarios, their training history. Task execution times are further influenced by the difficulty level assigned to the task and the nurse’s capability;
- Trainer Nurses—specialized agents assigned to train low-performing nurses in Scenario 3. They accompany the trainee during task execution, enabling skill improvement through repeated observation and guidance;
- Patients, who are dynamically spawned and assigned to beds, each requiring a specific task to be completed.

The specific logic governing how doctors determine task difficulty (evaluation style) and how nurse skill level affects task execution time (nurse quality) is described in detail as follows:

##### Doctor Evaluation Styles

In the simulation, each doctor is assigned a predefined evaluation style at the start of the shift. The evaluation style determines how the doctor estimates the performance level required to perform a specific task after examining a patient.

Three styles are implemented:

- Estimates Correctly—the doctor accurately assesses the true performance level;
- Overestimates—the doctor systematically assigns higher performance levels than the true value;
- Underestimates—the doctor systematically assigns lower performance levels than the true value.

When a doctor examines a patient, the function EvaluatePerformanceLevel() maps the task’s true difficulty to the estimated level according to the doctor’s evaluation style. The pseudocode of the core decision logic is presented in Algorithm A1 (see Appendix A.1). This design enables the simulation to model different diagnostic behaviors, allowing us to study how misestimating of task difficulty affects nurse decision-making, workload distribution, and overall system performance.

Although these evaluation styles are predefined in the current implementation, in a real-world emergency room, such decision-making processes may vary considerably across individual doctors and over time. Approaches for learning personalized evaluation functions from empirical data, potentially using machine learning, are discussed in Section 6.

##### Nurse Quality Attributes

At the start of each simulation run, nurses are instantiated with predefined quality attributes, currently set to either high-performing or low-performing. This attribute determines their expected task execution time and, consequently, their likelihood of task success. Nurse performance is modeled in the GetTaskDuration() function. Here, the nurse’s quality influences the execution time for each task, with high-performing nurses generally completing tasks faster and with higher consistency, and low-performing nurses showing slower and more variable execution times. In training scenarios, low-performing nurses can improve their performance by performing tasks under the instructions of a trainer nurse, represented by an incremental bonus chance in their task duration calculation. The logic governing tasks execution speed for different nurse qualities is summarized in Algorithm A2 (see Appendix A.2).

Such an approach allows us to systematically simulate the impact of different nurse skill levels on task allocation, patient service times, and overall ER performance. However, as with doctor evaluation styles, in a real-world emergency room, nurse performance is influenced by a complex interplay of factors, including experience, training, fatigue, workload, and individual skill variations. In Section 6, we discuss the potential of using machine learning to learn this nurse performance model directly from empirical data, enabling the simulation to more accurately reflect real-world variability and adaptive behaviors.

By modeling distinct decision styles and skill levels for individual agents, the framework effectively simulates personalized behavioral variability—reflecting how real-world differences among clinicians can influence outcomes in precision medicine contexts.

It should be noted that, in the current implementation, agent decision processes are encoded through predefined, rule-based functions. This modeling choice was made intentionally to ensure experimental control and reproducibility when comparing alternative coordination policies. However, we acknowledge that such hard-coded behaviors do not fully capture the contextual variability and adaptive learning dynamics characteristic of real clinical environments. Future extensions of the framework will therefore integrate data-driven behavioral adaptation, allowing parameters such as trust thresholds, evaluation biases, and performance estimates to evolve over time based on logged experience or empirical data. This enhancement will strengthen the ecological validity of the simulation and align the model with the broader goal of AI-driven personalization in healthcare operations.

#### 3.1.3. ER Layout and Environment Setup

The emergency room (ER) is implemented as a structured 3D environment within Unity, designed to support realistic agent navigation and interactions.

**Figure 1:**
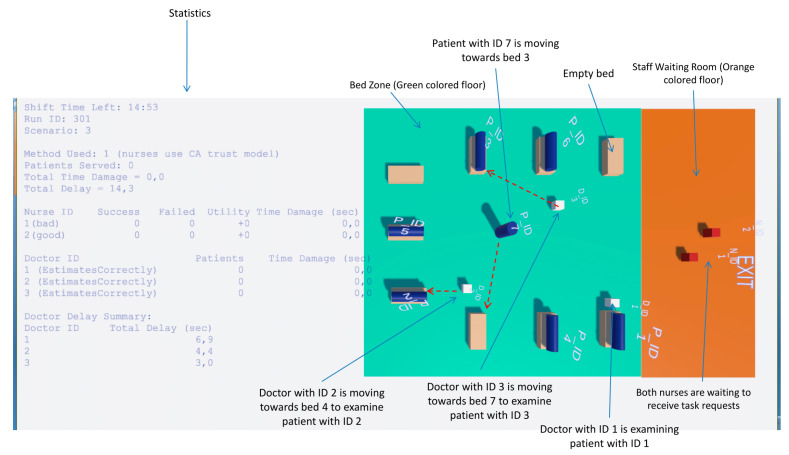
Simulation snapshot showing the bed zone (green area), staff waiting room (orange area), and the movement of agents. Red arrows indicate movement directions, while labels describe the current activity of agents. Dynamically updated statistics are displayed on the left side.

Figure 1 illustrates the layout, which includes the following key areas:

- Bed Zone—A green colored floor area where patient beds are located and treatments are performed. The ER contains nine available beds. Each doctor has exclusive access to a fixed set of beds: Doctor with ID 1 is assigned to Beds 1–3, Doctor with ID 2 to Beds 4–6, and Doctor with ID 3 to Beds 7–9. This allocation constrains patient assignments and task requests to the relevant doctor-bed pairs;
- Staff Waiting Room—An orange-colored designated area where doctors and nurses are initially spawned at the start of the simulation and remain until they are assigned to patient care in the Bed Zone. Nurses also return to this area when no pending tasks are available;
- ER Exit Point—A tagged location (ERExit) where agents, such as patients who have completed treatment, navigate when leaving the ER;
- UI Overlays—Floating labels and task timers are attached to agents to display real-time information such as agent IDs and remaining task durations.

While the current implementation adopts a fixed number of beds, a predefined spatial arrangement, and static bed allocations to doctors, possible extensions for dynamically generated ER layouts and site-specific operational workflows are discussed in Section 6.

#### 3.1.4. Scenarios and Decision Policies

The framework supports multiple configurable scenarios, each representing a different nurse behavior policy.

The active scenario is defined by two global parameters:

- GlobalSettings.methodToChooseRequest: specifies the strategy for task selection (e.g., CA trust model, FIFO);
- GlobalSettings.scenario:

Specifies high-level behavioral logic when nurses use the CA trust model:

1. Scenario 1—Baseline: low-performing nurses limit their participation to tasks below a certain difficulty threshold;
2. Scenario 2—Replacement: low-performing nurses request substitution;
3. Scenario 3—training with mentor: a trainer nurse assists the low-performing nurse, enabling performance improvement.

The modular scenario design allows easy extension for future behavioral studies. Such modularity also supports the implementation of personalized staffing and decision policies, where agent behaviors and task thresholds could be dynamically tuned to individual competencies or patient needs—a core requirement of personalized medicine.

#### 3.1.5. Workflow

##### Base Workflow

At the start of the simulation, patients are spawned one by one and assigned to available beds following a predefined sequence (Bed 1 → Bed 4 → Bed 7 → Bed 2 → Bed 5 → Bed 8 → Bed 3 → Bed 6 → Bed 9). This sequence ensures that all three doctors begin examining patients as early as possible. Once all beds are occupied, each subsequent patient is spawned only when a bed becomes free, immediately moving to occupy it and waiting to be examined by the corresponding doctor.

Each doctor leaves the staff waiting room as soon as the first patient lies down in one of their assigned beds. The doctor then moves next to the patient’s bed, performs an examination (simulated with a fixed delay of 10 s), and then issues task requests to nurses (e.g., insertion of an intravenous (IV) catheter).

In the current implementation, the workflow models only one type of nursing task—IV catheter insertion. Extensions to include a broader range of nursing interventions are discussed in Section 6.

Nurses evaluate incoming requests based on their assigned decision-making policy and decide whether to execute or ignore them. When nurses accept a task, they move to the assigned patient, verify whether the task has already been completed by another nurse, and execute it if still pending. Nurses do not currently coordinate with each other to share or delegate tasks; however, such collaborative behavior could be added in future scenarios.

Task execution is simulated with a time delay, during which a floating UI label above the nurse displays the remaining time. Once the task is complete, the nurse returns to the staff waiting room to prepare for the next task (e.g., washing hands, gathering necessary materials). While these preparatory activities are not yet visually represented in the simulation, they could be incorporated in future iterations to enhance realism.

Nurses remain idle in the waiting room either when no tasks are available, or when they have self-assessed as low-performing and no tasks suitable to their skill level exist.

##### Replacement Scenario Workflow

In Scenario 2 (Replacement), nurses self-classified as low-performing using the CA trust model remain active in patient care but restrict their participation to low-difficulty tasks as in the Baseline scenario. To prevent delays in executing these higher-difficulty tasks, the simulation spawns an additional high-performing nurse, who takes over the pending requests of the low-performing nurse. The replacement nurse starts from the staff waiting room and begins executing these tasks immediately.

This approach models a hospital policy in which underperforming staff are supplemented with additional personnel rather than replaced entirely, ensuring that difficult tasks are still addressed promptly while allowing the original nurse to continue contributing within their capability range.

##### Training Scenario Workflow

In Scenario 3 (training with mentor), when a nurse determines her performance is low-performing, she requests the assistance of a dedicated trainer nurse. This trainer is spawned near the underperforming nurse and follows her to observe the execution of tasks. The trainer’s role is to mentor the nurse through live task execution, improving her performance over time.

During the training phase, the trainee’s task performance gradually improves based on observed tasks, with each completed observation incrementing the learning progress. The trainer directly supervises the execution process but does not perform the tasks themselves. Once the trainee’s performance metrics surpass the required threshold, the training phase ends, the trainer is leaving ER, and the nurse resumes full responsibilities independently.

This scenario reflects real-world continuous knowledge transfer and skill improvement, where mentoring is provided as an alternative to staff replacement, aiming to restore full performance capabilities.

#### 3.1.6. Logging and Metrics

The simulation automatically tracks and logs detailed performance metrics per shift, including the following:

- Number of patients served—Measures the total number of patients who have been fully attended to during the shift. A patient is considered “served” when all tasks requested by their assigned doctor have been successfully performed by the nurses. In addition to the total shift value, this metric is also recorded per doctor, enabling analysis of how the doctor’s evaluation style (estimates correctly, overestimates, underestimates) relates to the number of patients served under their care;
- Total time damage—Quantifies the cumulative extra time taken by nurses to complete tasks beyond the expected task duration. This excess time is considered detrimental to patient outcomes—either by reducing the patient’s survival probability (e.g., in cases such as cardiac arrest where an IV catheter must be placed quickly to administer life-saving medication) or by increasing patient dissatisfaction due to prolonged waits. The simulation computes this metric by summing all positive deviations (actual duration—expected duration) for completed tasks. In addition to the overall value for each shift, time damage is recorded per nurse—enabling analysis of how a nurse’s quality attribute (e.g., high-performing, low-performing) correlates with the delays they cause—and per doctor, where it reflects the cumulative delays affecting that doctor’s patients. This doctor-level recording allows investigation of how a doctor’s evaluation style (estimates correctly, overestimates, underestimates) influences the delays, e.g., whether underestimation of task difficulty leads to higher time damage;
- Total delay—Measures the total amount of time patients remain unattended by any nurse after they have been examined by their assigned doctor and one or more tasks requests have been sent to nurses. These are pure waiting periods in which the patient occupies a bed but receives no active nursing intervention. In addition to the overall shift value, total delay is also recorded per doctor, allowing the analysis of how each doctor’s evaluation style influences the waiting times experienced by their patients;
- Task success/failure counts—For each nurse, the simulation records the number of successfully and unsuccessfully completed tasks. A task is counted as successful if the actual execution time does not exceed the time estimated and requested by the doctor who examined the patient. These metrics therefore represent the doctor’s assessment of each nurse’s performance in executing assigned tasks. As a result, due to a doctor’s systematic bias in evaluation style (see Section 3.1.2), a task may be labeled unsuccessful despite meeting the gold-standard duration (in cases of overestimation), or labeled successful despite exceeding it (in cases of underestimation). Overestimation occurs when the doctor assigns higher performance levels than required, imposing a stricter time threshold, while underestimation relaxes the threshold. For instance, if the true required level is 3 (40 s) but the doctor overestimates and requests level 5 (20 s), a completion time of 30 s—although below the true gold-standard—will be labeled unsuccessful. Conversely, underestimation may label the same 30 s as successful, despite exceeding the true optimal duration;
- Utility values for each nurse—For each nurse, the simulation records a cumulative utility score based on successfully completed tasks. When a task is successful (i.e., its actual execution time does not exceed the time estimated and requested by the examining doctor), the nurse’s utility increases by the task’s requestedLevel—an integer from 1 (easiest) to 5 (most difficult) representing the task’s difficulty. This metric can serve as a motivational indicator, with nurses potentially aiming to maximize their utility over the course of the shift;
- Doctors delays and evaluation accuracy.

These metrics are displayed in the in-game UI and exported to external .csv files for post-simulation statistical analysis. These performance indicators can also serve as proxies for evaluating personalized decision-support performance, linking individual agent behaviors with patient-level outcomes.

#### 3.1.7. Customizability and Reproducibility

The platform is designed for experimental flexibility:

- Agent counts, roles, and attributes are configured via the GlobalSettings class;
- Scenario-specific logic is encapsulated for modular extension;
- Run IDs and parameter logging ensure reproducibility of results.

This modular, data-driven design supports iterative development of new scenarios and facilitates rigorous comparative analysis.

### 3.2. Decision-Support Experimentation Methodology

#### 3.2.1. Objectives and Rationale

The primary aim of the decision-support experiments is to evaluate how alternative nurse decision-making policies and workflow strategies influence key operational outcomes in a simulated emergency room (ER) environment. By systematically varying agent behaviors and resource allocation strategies, the framework allows us to identify policies that minimize patient delays, reduce detrimental time damage, and optimize the utilization of available staff. Beyond operational benchmarking, the proposed framework provides a foundation for AI-driven personalization of clinical workflows, allowing the adaptation of decision policies to specific staff profiles and patient characteristics.

This approach supports data-driven decision-making in hospital management, enabling stakeholders to test “what-if” scenarios before implementing changes in real clinical settings.

#### 3.2.2. Experimental Design

##### Scenarios

Three distinct operational scenarios within the simulation framework can be evaluated. Each represents a different policy for handling low-performing nurses.

Scenario 1—Baseline: Low-performing nurses remain active but only perform tasks below a predefined difficulty threshold. More complex tasks are skipped by these nurses and remain unassigned until another available nurse chooses to execute them. Unlike the Replacement scenario, where an additional high-performing nurse assists with complex tasks, and the Training scenario, where a trainer nurse accompanies and guides the low-performing nurse, no extra nursing staff is provided in the Baseline scenario.

Scenario 2—Replacement: Low-performing nurses remain active but only perform tasks below a certain difficulty threshold; and additional high-performing nurse is spawned to execute pending higher-difficulty tasks, ensuring they are addressed without excessive delay.

Scenario 3—Training with Mentor: Low performing nurses are accompanied by a trainer nurse who supervises and guides them, improving their performance over time.

It is important to note that the FIFO decision-making policy is only meaningful in the Baseline scenario, as it does not support performance self-assessment or adaptive responses to low quality. In contrast, the CA trust model is applicable across all scenarios and is essential in Replacement and Training settings, as it enables nurses to detect low performance and trigger the corresponding assistance or mentoring mechanisms.

##### Controlled Variables

- Number of doctors, and nurses;
- ER layout and bed allocation rules;
- Patient assignment sequence—fixed order in which patients occupy available beds to maximize early doctor engagement (1 → 4 → 7 → 2 → …);
- Task types;
- Evaluation styles of doctors and quality attributes of nurses (unless explicitly varied).

##### Independent Variables

- Nurse decision-making policy (CA trust model, FIFO);
- Scenario type (Baseline, Replacement, Training);
- Doctor evaluation styles (overestimates, underestimates, estimates correctly);
- Nurse quality attributes (high-performing, low-performing).

##### Dependent Variables

The simulation tracks a set of predefined performance metrics (see Section 3.1.6), including:

- Number of patients served;
- Total delay;
- Total time damage;
- Task success/failure counts;
- Utility values per nurse.

#### 3.2.3. Experimental Procedure

The experiments are conducted for four specific scenario-policy combinations:

1. Baseline scenario—CA trust model;
2. Baseline scenario—FIFO;
3. Replacement scenario—CA trust model;
4. Training scenario—CA trust model.

For each of these combinations, the simulation is executed 60 times to account for stochastic variations in task durations and agent behaviors, with different random seeds to ensure statistical robustness. In the Replacement and Training scenarios, only the CA trust model is tested, as this decision-making policy is the only one that allows nurses to detect their low performance and either request assistance from an additional high-performing nurse (Replacement) or receive guidance from a trainer nurse (Training). The FIFO policy is applied only in the Baseline scenario.

Simulation parameters are initialized via the GlobalSettings class. A unique Run ID is assigned to each execution for reproducibility. Patient spawning, doctors’ examinations, and nurse tasks executions proceed according to the workflows described in Section 3.1.5. Performance metrics are automatically logged both in the in-game UI and in external CSV files.

#### 3.2.4. Data Analysis Approach

Collected data are analyzed using descriptive statistics and inferential statistical tests (e.g., ANOVA, *t*-tests, or non-parametric equivalents where appropriate) to determine whether observed differences between policies are statistically significant.

From a decision-support perspective, the results highlight trade-offs between different strategies. For instance, the Replacement scenario may significantly reduce total delay for complex tasks but could require additional staffing resources, while the Training scenario may offer long-term benefits by improving low-performing nurses without permanently increasing headcount.

This analysis framework enables hospital managers and policymakers to

1. Predict the operational impact of specific interventions before real-world deployment;
2. Compare alternative policies under controlled and repeatable conditions;
3. Identify scenario-specific strengths and weaknesses, informing adaptive staffing and training strategies.

The analytical framework further enables the exploration of personalized decision patterns, where performance metrics are examined at the level of individual agents, highlighting how staff-specific adaptation can contribute to precision medicine initiatives.

## 4. Results

### 4.1. Case Study Example

To illustrate the practical use of the simulation framework for decision support, we present a case study of a small emergency room (ER) setup. The configuration includes three doctors (all accurately estimating task requests), two nurses (one high-performing and one low-performing), and nine beds.

Three main interventions were compared across multiple simulation runs:

1. Baseline, comparing task allocation methods (CA trust model vs. FIFO);
2. Replacement, introducing an additional high-performing nurse for difficult tasks;
3. Training, pairing the low-performing nurse with a trainer to improve skills over time.

A full methodological description, including detailed statistical analyses, is provided in the Supplementary Materials (Decision Support Case Study). In this section, we focus only on the key findings, their interpretation, and the main limitations, along with the conclusions drawn. This case study also illustrates how individualized staff behaviors and adaptive coordination strategies can be represented computationally, providing a bridge toward personalized and precision-oriented decision support in emergency care.

### 4.2. Key Findings

**Baseline comparison (CA trust vs. FIFO)**: The FIFO policy served more patients and reduced overall waiting times, but substantially increased patient damage time and task failures by the low-performing nurse. In contrast, the CA trust model prioritized safety, reducing harmful delays and error rates but at the cost of longer waiting times. This demonstrates the trade-off between safety and operational efficiency. From a personalized medicine perspective, this trade-off reflects the dynamic balance between individual performance reliability and patient-specific safety outcomes, highlighting the importance of adaptive task allocation.

**Replacement Scenario**: Adding an extra high-performing nurse to handle complex tasks significantly improved patient throughput and reduced delays, achieving faster service and smoother patient flow. However, the increase in staff resources slightly raised cumulative patient damage time and implied additional staffing costs.

**Training Scenario**: Pairing the low-performing nurse with a trainer improved throughput and led to gradual skill gains. While error rates initially increased—resulting in higher short-term patient damage—the overall system performance stabilized over time, reflecting the long-term benefits of capability building.

**Replacement vs. Training**: Replacement produced immediate gains in efficiency and patient flow, whereas Training supported gradual skill development of low-performing nurses. The decision between these strategies depends on managerial priorities: short-term operational optimization versus long-term workforce development. In a broader sense, these contrasting strategies represent complementary dimensions of personalized decision-making—one emphasizing immediate, patient-level optimization (Replacement) and the other focusing on long-term personalization of staff performance (Training).

### 4.3. Interpretation and Limitations

This case study is based on a specific configuration of doctors, nurses, and beds. Results may vary significantly under different conditions—for example, if doctor evaluation styles differ (overestimating or underestimating tasks) or if the nurse composition changes (e.g., two high-performing nurses). Additionally, the type of task being simulated can strongly influence outcomes. In this study, IV catheter insertion was used—a task where improvement of low-performing nurses occurs gradually over time in the Training scenario. For other tasks, such as vital sign measurement, improvement may occur rapidly. In such cases, even a single demonstration might suffice for a low-performing nurse to reach a high performance, potentially making the Training scenario more effective than Replacement across all performance indicators for that task. Therefore, the findings should be interpreted as illustrative of the framework’s capabilities rather than universally generalizable conclusions. Nevertheless, the ability to parameterize agent-level variability—such as doctor judgment bias or nurse learning curves—positions the framework as a foundation for data-driven personalization of decision-support models in clinical operations.

### 4.4. Summary of Findings

The case study highlights how the simulation framework supports evidence-based decision-making by allowing hospital managers to explore trade-offs between patient safety, operational efficiency, and staff development under controlled, repeatable conditions. This demonstrates that policy selection depends on strategic priorities, and highlights the potential of the framework as a decision-support tool to test alternative scenarios before real-world implementation. Moreover, by integrating adaptive trust-based coordination with individualized agent modeling, the framework contributes to the emerging vision of AI-driven personalized medicine, where operational decisions are continuously tuned to both staff performance and patient-specific needs.

## 5. Discussion

The results of our simulation experiments demonstrate the potential of trust-based allocation mechanisms to enhance decision-making in emergency department (ED) staffing and workflow coordination. By comparing baseline, replacement and training scenarios under both FIFO and CA trust-based decision policies, our study highlights key trade-offs between patient safety, operational efficiency, and staff development.

From an artificial intelligence perspective, the proposed simulation can be viewed as an adaptive multi-agent system in which each agent continuously updates its behavior based on contextual feedback and inferred performance levels. This aligns with the principles of personalized healthcare operation, where decision-making is dynamically tailored to both individual staff capabilities and patient-specific needs.

A central finding is that the CA trust model consistently prioritizes patient safety by detecting low-performing nurses and limiting their exposure to high-complexity tasks. This reduces the likelihood of critical errors but introduces longer delays, especially in the baseline scenario. In contrast, the FIFO policy improves fairness and reduces waiting times but exposes patients to greater risks associated with incorrect or incomplete task execution. This contrast underscores the inherent tension between safety-oriented and efficiency-oriented policies in high-pressure ED environments.

The introduction of replacement and training interventions provides complementary strategies for mitigating the limitations of the baseline configuration. The replacement scenario offers immediate operational benefits by assigning high-complexity tasks to an additional high-performing nurse, thereby reducing both patient delays and cumulative time damage. However, these gains come with increased resource demands and higher operational costs, which may not be sustainable under staffing or budgetary constraints. While the Replacement scenario conceptually considers the financial implications of adding staff, these effects are not explicitly quantified in the current study. Future work could integrate cost modeling to assess the economic trade-offs of staffing interventions, thereby strengthening the framework’s utility as a decision-support tool. Incorporating financial metrics alongside operational and patient-centered outcomes would provide a more comprehensive basis for evaluating alternative staffing strategies. The training scenario, by contrast, provides a mechanism for capacity and capability building, enhancing not only the system’s overall throughput but also the skill and adaptability of individual agents: low-performing nurses gradually improve through guided practice with a trainer nurse. While this incurs short-term delays and higher patient risk, it fosters long-term workforce development and reduces reliance on permanent staffing increases. Together, these findings suggest that replacement and training are not mutually exclusive but may be strategically combined depending on institutional priorities and resource availability.

From a broader perspective, our findings contribute to the growing literature on multi-agent trust and reputation systems by demonstrating their applicability to healthcare operations. While trust-based models have been widely studied in domains such as online marketplaces, peer-to-peer systems, and the Internet of Things, their integration into healthcare workforce management remains underexplored. In this context, computational trust operates analogously to AI-driven personalization, serving as a mechanism through which agents modulate collaboration intensity and task allocation according to learned reliability patterns—effectively mirroring how clinical AI systems personalize treatment decisions based on individual patient data. By modeling nurses and doctors as intelligent agents capable of self-assessment and adaptive decision-making, our framework extends computational trust research into a domain where the stakes of decision errors are exceptionally high.

The case study analysis further illustrates how the framework can support hospital managers in evidence-based decision-making. For example, a small ED with limited staff may initially favor replacement strategies to ensure patient throughput, but transition to training strategies once capacity constraints are stabilized. By offering a sandbox for “what-if” experimentation, the framework allows decision-makers to explore the consequences of operational policies before implementation, reducing the risks of trial-and-error approaches in real clinical settings.

Nevertheless, the study has several limitations that must be acknowledged. First, the results are derived from a restricted configuration involving a single, small-scale case study with predetermined numbers of doctors, nurses, and tasks, which limits their direct generalizability and constrains external validity. While this controlled setup enables clear observation of causal relationships and model behavior, future studies should replicate the framework across multiple emergency departments with varying sizes, staffing levels, and patient acuity distributions to strengthen generalizability and practical relevance. Second, the current implementation models only a single nursing task—IV catheter insertion. This deliberate simplification ensures experimental control and allows clear observation of how trust-based coordination mechanisms influence safety, efficiency, and training outcomes. In this sense, the current implementation serves as a proof of concept, demonstrating the framework’s capability to model trust-based decision-making dynamics under controlled conditions. However, real-world emergency departments involve a broad spectrum of nursing procedures that vary in complexity, frequency, and required expertise. Extending the framework to include multiple task types (e.g., medication administration, vital sign measurement, or ECG recording) would provide a richer and more realistic representation of clinical workflows, improving the generalizability of staffing strategy insights. Finally, agent behaviors are rule-based, meaning that contextual variability, learning effects, and human factors such as fatigue or stress are not yet represented. In future iterations, incorporating machine learning-based behavioral adaptation could allow agents to evolve performance profiles over time, enabling deeper personalization of both staff and patient interactions. Moreover, several key operational factors that influence real ED performance—such as staff fatigue, shift transitions, multi-tasking demands, and the mix of patient acuity—are not yet modeled in the current framework. Incorporating these aspects in future work would enhance ecological validity and allow the simulation to more accurately reflect the dynamic and high-pressure nature of emergency care. For example, modeling fatigue and shift changes could influence agent performance over time, while including multi-tasking and diverse patient acuity could affect task prioritization and workflow efficiency. These constraints suggest that the findings should be interpreted as illustrative of the framework’s capabilities rather than prescriptive for real-world decision-making.

Looking ahead, the framework provides a foundation for more adaptive and realistic modeling of ED operations. As outlined in Section 6, integrating data-driven behavioral models, customizing ED environments to reflect site-specific layouts, and expanding the range of nursing tasks will significantly increase ecological validity. Such enhancements will strengthen the framework’s role as a practical decision-support tool, enabling hospital managers to balance short-term efficiency with long-term workforce sustainability.

In summary, the present work highlights the importance of trust-based allocation mechanisms in managing uncertainty and variability in emergency care. By explicitly modeling the trade-offs between safety, efficiency, and staff development, our framework offers both theoretical insights into computational trust models and practical decision-support value for healthcare institutions. Beyond its operational insights, the framework demonstrates how AI-enabled trust modeling can contribute to the broader vision of personalized, data-driven healthcare, where resource allocation and care delivery adapt dynamically to the unique characteristics of both patients and medical staff.

## 6. Conclusions and Future Work

The proposed framework illustrates the potential of computational trust as a foundation for personalized and evidence-based decision support in emergency medicine. By integrating adaptive and AI-informed coordination mechanisms, it enables hospital managers to evaluate alternative staffing and treatment strategies under controlled and reproducible conditions. In doing so, the framework bridges operational optimization with the broader goals of precision and personalized medicine, demonstrating how resource allocation and care delivery can dynamically adapt to both patient needs and staff capabilities. This proof-of-concept study thereby establishes a groundwork for future AI-driven, data-informed decision support systems in healthcare.

Having demonstrated the applicability of our simulation framework for modeling staff coordination and trust-based task allocation in an emergency department (ED) setting, we envision several directions for future research. These directions aim to increase the realism of agent behavior, to better capture the variability of real-world emergency workflows, and to expand the decision-support capabilities of the system. By moving beyond fixed, rule-based representations, the framework can evolve into a more adaptive platform capable of supporting evidence-based policy evaluation and operational planning.

### 6.1. Learning Agent Behavior Models from Logged Performance Data

In the current implementation, both doctor evaluation styles and nurse quality attributes are modeled through fixed, rule-based functions (see Section 3.1.2). While this design enables controlled comparisons between scenarios, it does not capture the variability and context-dependent decision-making that characterize real ED staff.

A promising extension is to replace these hard-coded rules with data-driven models inferred from the simulation’s own detailed performance logs or from real-world clinical data, integrating machine learning components to capture behavioral variability and allow agents to continuously adapt their decision strategies to evolving clinical contexts—a key feature of AI-driven personalization.

#### 6.1.1. Inferring Doctor Evaluation Styles

Doctor-level logs include total patient time damage, cumulative delays before treatment, and the number of patients served. Analyzing the relationships among these metrics could allow inference of doctor evaluation styles. For instance, systematic underestimation of task difficulty may correlate with increased patient damage, while overestimation may inflate perceived nurse error rates. Machine learning methods such as classification, clustering, or Bayesian inference could estimate probability distributions over evaluation styles, leading to more realistic physician-agent behaviors.

#### 6.1.2. Inferring Nurse Quality Attributes

Nurse-level logs record task outcomes (success/failure counts), time damage, delay impacts, and utility values. While the current framework categorizes nurses as simply high-performing or low-performing, this binary classification oversimplifies reality. Regression models, clustering approaches, or reinforcement learning could be employed to infer richer nurse skill profiles, capturing variability due to experience, fatigue, training, or workload.

By adopting this dual-level learning approach, the framework could evolve into a self-adaptive platform in which agent parameters are continuously updated based on observed behavior, supporting more accurate predictive modeling and more credible decision-support analyses.

### 6.2. Environment and Workflow Customization

Future extensions should allow the emergency department (ED) environment to be dynamically configured based on hospital-specific layouts, staffing structures, and operational policies. Incorporating digital twins or blueprints would enable site-specific replication of bed capacity, nurse stations, and patient flow paths, enhancing the ecological validity of the simulation and its value as a decision-support tool.

### 6.3. Integrating a Triage Mechanism for Task Prioritization

Although the current simulation framework does not explicitly include a triage module, aspects of triage are implicitly represented through the doctor’s evaluation of required performance levels, which reflect both patient acuity and task complexity. This implicit mechanism ensures that more demanding or urgent tasks are indirectly associated with higher performance requirements. However, the current CA algorithm does not prioritize tasks according to case severity or patient urgency. Future extensions of the framework could incorporate an explicit triage mechanism that dynamically orders tasks based on patient acuity and clinical urgency.

### 6.4. Expanding the Range of Nursing Tasks

This current framework models only one nursing intervention (IV catheter insertion). Extending it to include a wider range of routine ED tasks—such as medication administration, vital signs measurement, or ECG—would increase realism and enable comparative evaluation of staff allocation strategies. This would also allow hospital managers to explore how Replacement and Training policies perform across different task complexities, balancing efficiency with long-term workforce development.

This study highlights the potential of computational trust models to transform how we understand and optimize emergency department operations. By integrating trust-driven coordination into agent-based simulations, we bridge the gap between individual decision-making, team performance, and patient-centered outcomes. Looking ahead, trust-aware mechanisms could form the basis for adaptive, AI-driven systems that not only improve efficiency but also enable personalized, context-aware models of care. By linking computational trust with predictive learning the framework provides a foundation for precision decision support—aligning operational optimization with the broader goals of personalized medicine.

## Data Availability

The original data presented in the study are openly available in the Supplementary Materials.

## Supplementary Materials

The following supporting information can be downloaded at https://www.mdpi.com/article/10.3390/jpm15120574/s1, Table S1: simulation results; File S1: RunLogs-Zip; File S2: Decision Support Case Study.

## Appendix A. Algorithms

### Appendix A.1

**Algorithm A1:** Doctor’s performance level estimation based on evaluation style.
function EvaluatePerformanceLevel(trueLevel, evaluationStyle):
switch(evaluationStyle):
case “overestimates”:
if trueLevel <= 2: return 3
if trueLevel >= 3: return 5

case “underestimates”:
if trueLevel >= 4: return 3
if trueLevel <= 3: return 1

case “estimatesCorrectly”:
return trueLevel
default:
return trueLevel

### Appendix A.2

**Algorithm A2:** Nurse task execution time determination based on quality and training status.
function GetTaskDuration(nurseId, truePerformanceLevel, observedTasks):
roll = random(0, 1)

if nurseId == 1: // Low-performing nurse
isTrainingScenario = (methodToChooseRequest == 1 AND Scenario == 3)

if isTrainingScenario:
bonusChance = clamp(observedTasks × 0.1, 0, 1) // each observed task adds 10%

if roll <= bonusChance:
return baseDurationForLevel(truePerformanceLevel)
else:
return randomDurationAboveBase(truePerformanceLevel)
else:
return randomDurationAboveBase(truePerformanceLevel)

else if nurseId == 2: // High-performing nurse
rollGood = random(0, 1)

if rollGood <= 0.90:
return baseDurationForLevel(truePerformanceLevel)
else:
return randomDurationAboveBase(truePerformanceLevel)

return 40 // default fallback

function baseDurationForLevel(level):
switch(level):
case 1: return 60
case 2: return 50
case 3: return 40
case 4: return 30
case 5: return 20
default: return 40

function randomDurationAboveBase(level):
// returns a duration in a higher range to simulate delay
switch(level):
case 1: return random(60, 70)
case 2: return random(50, 70)
case 3: return random(40, 50)
case 4: return random(30, 40)
case 5: return random(20, 30)
default: return 40
