# Supplementary material for: From Coordination to Personalization: A Trust-Aware Simulation Framework for AI-Driven Personalized Decision Support in Emergency Departments
Source: J Pers Med. 2025 Nov 28;15(12):574. doi: 10.3390/jpm15120574 (PMC12734260; doi:10.3390/jpm15120574)
Supplement: Supplementary file 1 [file jpm-15-00574-s001.zip › File S2 - Decision Support Case Study.pdf]

Decision Support Case Study

This supplementary material provides full methodological and statistical details for the decision-support case study summarized in Section 4 of the main manuscript. All data correspond to 60-run simulation averages and include statistical test results (Shapiro-Wilk, Wilcoxon rank-sum, Monte Carlo x^2, etc.).

B.1. Comparative Evaluation of Nurse Task Selection Methods in the Baseline Scenario

Objective

This case study investigates how two alternative nurse task selection policies affect patient care quality and operational performance metrics in the Baseline scenario:

- Method 1: CA trust model;
- Method 2: FIFO.

Design

- Scenario: Baseline (no replacement nurses, no training interventions);
- Task selection policies: CA trust model vs. FIFO;
- Simulation runs: 60 independent runs per method;
- Metrics analyzed: Total patients served, total patient damage time, total patient delay, failed and successful tasks per nurse, nurse utility, nurse time damage, patient distribution across doctors.

Statistical Approach

All metrics were tested for normality using the Shapiro-Wilk test. Due to significant deviations from normality in most cases (p<0.001), non-parametric Wilcoxon rank-sum tests were applied unless otherwise specified. Effect sizes, medians, means and p-values are reported for each comparison.

Key Results

Total Patients Served: Since both groups exhibited significant deviations from normality (Shapiro Wilk p<0.001), the non-parametric Wilcoxon rank-sum test was applied. The analysis showed that the FIFO policy served significantly more patients than the CA trust model ($W = 0, p < 2.2 \times 10^{-16}$). The mean difference was approximately 11 patients per shift (CA trust: 22.18, FIFO: 33.22), indicating that prioritizing tasks by arrival order rather than trust scores substantially increases throughput in the Baseline scenario.

Total Patient Damage Time: Both groups deviated significantly from normality (Shapiro-Wilk p<0.001), so the non-parametric Wilcoxon rank-sum test was applied. The CA trust model resulted in dramatically lower cumulative patient damage time compared to the FIFO policy ($W = 0, p < 2.2 \times 10^{-16}$). On average, the damage time was reduced by nearly 200 seconds per shift (CA trust: 65.38 s, FIFO: 262.77 s),

indicating that trust-based task allocation substantially mitigates patient exposure to harmful delays in treatment.

Total Patient Delay: Both groups showed strong deviations from normality (Shapiro-Wilk p<0.001), so the Wilcoxon rank-sum test was used. FIFO produced significantly lower total patient delay compared to the CA trust model ($W = 90000, p < 2.2 \times 10^{-1}$). The mean reduction was about 642 seconds per shift (CA trust: 7095.77 s, FIFO: 6453.23 s), suggesting that FIFO's task allocation leads to shorter waiting periods for patients before nursing intervention. However, as earlier results show, this comes at the cost of increased patient damage time.

Failed Tasks by the Low-performing Nurse: Both groups exhibited strong deviations from normality (Shapiro-Wilk, p<0.001), so the non-parametric Wilcoxon rank-sum test was applied. Results showed that the low-performing nurse failed significantly more tasks under FIFO (Method 2) than under the CA trust model (Method 1) ($W = 3600, p < 2.2 \times 10^{-16}$). The mean failure count was 13.53 for Method 2 and 3.60 for Method 1, a difference of nearly +10 failed tasks per shift. This confirms that FIFO's inability to avoid high-difficulty tasks for low-performing nurses leads to a substantial increase in failed task executions.

Failed Tasks by the High-performing Nurse: Normality tests indicated significant deviations from the normal distribution in both groups (Shapiro-Wilk p<0.001), so the Wilcoxon rank-sum test was used. The test did not reveal a statistical significant difference in failed tasks between FIFO and the CA trust model ($W = 1852.5, p = 0.389$). Mean failure counts were very similar (Method 1: 1.92, Method 2: 1.98), and the distributions showed substantial overlap. These results suggest that high-performing nurses maintain a low and stable error rate regardless of the task allocation policy.

Successful Tasks by the Low-performing Nurse: Both groups contained only zero values for the number of successful tasks, making normality testing infeasible and rendering statistical comparisons trivial. The Wilcoxon rank-sum test confirmed no difference between Method 1 (CA trust model) and Method 2 (FIFO) ($W = 1800, p = 1$). In other words, low-performing nurses were unable to complete any tasks successfully under either task allocation policy, highlighting that policy choice alone does not improve task success when nurse performance is intrinsically poor.

Successful Tasks by the High-performing Nurse: Shapiro-Wilk tests indicated that Method 1 data significantly deviated from normality (p<0.001), while Method 2 data showed no significant deviation (p=0.075). Given the distributional mismatch, the non-parametric Wilcoxon rank-sum test was applied. Results showed no significant difference in the number of successful tasks between Method 1 (median = 17) and

Method 2 (median = 18) ($W = 1853, p = 0.391$). These findings suggest that highly skilled nurses maintain similar success rates regardless of whether patient allocation follows the CA trust model or a FIFO policy.

Nurse Utility by the Low-performing Nurse: Shapiro-Wilk tests indicated that Method 1 data significantly deviated from normality (p=0.006), while Method 2 data showed no significant deviation (p=0.517). Given the distributional mismatch, the non-parametric Wilcoxon rank-sum test was applied. Results showed that the low-performing nurse had significantly lower utility in Method 2 (median = -44.50) compared to Method 1 (median = -9.97) ($W = 0, p < 2.2 \times 10^{-1}$). These findings suggest that the low-performing nurse performs substantially worse under Method 2 conditions.

Nurse Utility by the High-performing Nurse: Shapiro-Wilk tests indicated that Method 1 data significantly deviated from normality (p=0.027), while Method 2 data showed no significant deviation (p=0.916). Given the distributional mismatch, the non-parametric Wilcoxon rank-sum test was applied. Results showed no significant difference in nurse utility between Method 1 (median = 50.28) and Method 2 (median = 53.25) (W=1749, p=0.607). These findings suggest that highly skilled nurses maintain similar utility regardless of whether patient allocation follows Method 1 or Method 2.

Nurse Time Damage by the Low-performing Nurse: Shapiro-Wilk tests indicated that Method 1 data significantly deviate from normality (p=0.002), while Method 2 data showed no significant deviation (p=0.582). Given the distributional mismatch, the non-parametric Wilcoxon rank-sum test was applied. Results showed a significant difference in NurseTimeDamage between Method 1 (median = 56.48) and Method 2 (median = 252.37) ($W = 0, p < 2.2 \times 10^{-16}$). These findings suggest that low-performing nurses cause substantially lower time damage when patient allocation follows Method 1 compared to Method 2.

Nurse Time Damage by the High-performing Nurse: Shapiro-Wilk tests indicated that NurseTimeDamage data for both Method 1 (p=0.003) and Method 2 (p=0.003) significantly deviated from normality. Given the non-normal distributions, the non-parametric Wilcoxon rank-sum test was applied. Results showed no significant difference in NurseTimeDamage between Method 1 (median = 8.87) and Method 2 (median = 10.32) (W=1679, p=0.263). These findings suggest that highly skilled nurses maintain similar time damage regardless of whether patient allocation follows Method 1 and Method 2.

Patient Distribution Across Doctors: Nurses prefer some doctors in Method 1, while patients are served uniformly in Method 2. Using the Monte Carlo x^2 test for each simulation run, in Method 1, 18 out of 60 runs showed a statistically

significant nurse preference for certain doctors ($p<0.05$). In contrast, in Method 2, none of the runs showed a significant preference. The variance in the number of patients served per doctor per run was much higher in Method 1 (mean variance = 21.44) compared to Method 2 (mean variance = 0.6). A paired t-test confirmed that this difference is statistically significant $p \approx 1.46e-09$). The conclusion is that nurses appear to favor certain doctors in Method 1, resulting in an uneven distribution of patients. In Method 2, patients are distributed uniformly across doctors, as expected.

Conclusions

Method 1 (CA trust model) prioritizes patient safety and reduces the risk from low-performing nurses but can result in longer patient delays and potential doctor preference bias.

Method 2 (FIFO) improves fairness and reduces delays but increases exposure to nurse-related damage.

These results highlight a trade-off between safety and operational efficiency. Hospitals can select the appropriate method depending on their priorities: safety-focused institutions may prefer Method 1, while those emphasizing throughput and fairness may prefer Method 2.

B.2. Baseline (CA trust model) vs. Replacement (CA trust model)

Objective

Evaluate the impact of introducing a high-performing replacement nurse for tasks exceeding the difficulty threshold of low-performing nurses.

Design

- Scenarios: Baseline vs. Replacement;
- Task selection policy: CA trust model (applied in both scenarios);
- Metrics analyzed: Total patients served, total patient damage time, total patient delay, failed and successful tasks per nurse, nurse utility, nurse time damage, patient distribution across doctors.

Statistical Approach

All metrics were tested for normality using the Shapiro-Wilk test. Due to significant deviations from normality in most cases, non-parametric Wilcoxon rank-sum tests were applied. For metrics with no variance, no statistical test was meaningful. Effect sizes, medians, means, and p-values are reported for each comparison.

Key Results

Total Patients Served: Since both groups exhibited significant deviations from normality (Shapiro-Wilk $p<2.2e-16$), the non-parametric Wilcoxon rank-sum test was applied. Results showed that the Replacement scenario served significantly more patients $(W = 1935, p < 2.2 \times 10^{-16})$ compared to the baseline. The mean difference was

approximately 19.4 patients per shift (Baseline: 22.18, Replacement: 41.58), indicating that adding an extra nurse to handle high-difficulty tasks substantially increases the overall patient throughput.

Total Patient Damage Time: Since both groups showed significant deviations from normality (Shapiro-Wilk test: Baseline $p = 7.29 \times 10^{-9}$, Replacement $p = 1.63 \times 10^{-4}$, the non-parametric Wilcoxon rank-sum test was applied. Results revealed that the Replacement scenario had a significantly higher total time in damage (W=27360, $p < 2.2 \times 10^{-16}$) compared to the Baseline. The mean difference was approximately 30.6 seconds per shift (Baseline: 65.38 sec, Replacement: 95.97 sec), indicating that the staffing change, while improving throughput, also increased the cumulative time that patients spent in a critical condition.

Total Patient Delay: Shapiro-Wilk tests indicated that both the Baseline (CA trust) data (W=0.787, p<0.001) and the Replacement (CA trust) data (W=0.652, p<0.001) significantly deviated from normality. Given the non-normal distributions, the non-parametric Wilcoxon rank-sum test was applied, revealing a significant difference in total patient delay between the two scenarios ($W = 106605$, $p < 0.001$). Specifically, the Replacement scenario exhibited a substantially lower mean total delay (M=6284.12 s) compared to the Baseline scenario (M=7095.77 s) representing an average reduction of approximately 811.65 seconds per shift. These findings suggest that adding an extra nurse to handle high-difficulty tasks significantly decreases overall patient delay in the emergency room.

Failed Tasks by the Low-performing nurse: Since both groups exhibited significant deviations from normality (Shapiro-Wilk p<0.001), the non-parametric Wilcoxon rank-sum test was applied. Results indicated a small difference in the number of failed tasks between the Baseline and Replacement scenarios (W=1416.5, p=0.037), with the mean number of failed tasks being slightly higher in the Replacement scenario (Baseline: 3.6, Replacement: 4.0). This suggests that adding an extra nurse for high-difficulty tasks did not substantially reduce failed tasks, and the practical difference between the two scenarios is minimal.

Failed Tasks by the High-performing nurse: Since both groups exhibited significant deviations from normality (Shapiro-Wilk p<0.001), the non-parametric Wilcoxon rank-sum test was applied. Results showed no statistically significant difference in the number of failed tasks between the Baseline and the Replacement scenario (W=1744.5, p=0.7642). The mean number of failed tasks was identical across both scenarios (Baseline: 1.92, Replacement: 1.92), indicating that replacing one nurse did not affect the failure rate of high-performing nurse.

Successful Tasks by the Low-performing nurse: Since both groups exhibited no variance (all values were identical at 0), neither the Shapiro-Wilk test for normality nor inferential statistical tests (Wilcoxon, Welch's t-test) could be meaningfully applied. Both the Baseline and Replacement scenarios resulted in a mean of 0 successful tasks per shift, indicating that the low-performing nurse was unable to complete any successful tasks under either condition.

Successful tasks by the High-performing nurse: Since both groups exhibited significant deviations from normality (Shapiro-Wilk $p<0.001$), the non-parametric Wilcoxon rank-sum test was applied. Results showed no significant difference between the Baseline and Replacement scenarios ($W=1566.5$, $p=0.218$). The mean difference was approximately 1.25 successful tasks (Baseline: 16.67, Replacement: 17.92), indicating that the high-performing nurse achieved a comparable number of successful tasks in both conditions.

Nurse Utility by the Low-performing nurse: Since both groups exhibited significant deviations from normality (Shapiro-Wilk $p=0.006$ for Baseline, $p = 0.0009$ for Replacement), the non-parametric Wilcoxon rank-sum test was applied. Results showed that the Replacement scenario did not differ significantly in Nurse Utility of the low-performing nurse compared to the Baseline ($W= 1606$, $p=0.154$). The mean Nurse Utility was slightly lower for Replacement (-10.47) than for Baseline (-9.97), with a mean difference of approximately 0.5 units per nurse, indicating that replacing the low-performing nurse in Scenario 2 did not substantially change the overall nurse utility under Method 1.

Nurse Utility by the High-performing Nurse: Since both groups showed deviations from normality (Shapiro-Wilk $p<0.05$ for both Baseline and Replacement), the non-parametric Wilcoxon rank-sum test was applied. Results indicated that the Replacement scenario had significantly higher nurse utility ($W=2129.5$, $p=0.042$) compared to the Baseline. The mean difference was approximately 6.17 units of utility (Baseline: 50.28, Replacement: 56.45) suggesting that the Replacement scenario improves the efficiency of the high-performing nurse in Method 1.

Nurse Time Damage by the Low-performing Nurse: Since both groups exhibited significant deviations from normality (Shapiro-Wilk test, Baseline: $W=0.930$, $p=0.0021$, Replacement: $W=0.938$, $p=0.0045$), the non-parametric Wilcoxon rank-sum test was applied. Results showed no significant difference between Baseline and Replacement ($W=1955.5$, $p=0.794$), indicating that the time damage caused by the low-performing nurse remained similar across scenarios. The mean NurseTimeDamage was slightly higher in the Replacement scenario (Baseline: 56.48, Replacement: 58.07), but this difference was not statistically

significant. Thus, replacing the nurse team configuration did not reduce the time damage caused by the low-performing nurse.

Nurse Time Damage by the High-performing Nurse: Since both groups exhibited significant deviations from normality (Shapiro-Wilk $p<0.05$), the non-parametric Wilcoxon rank-sum test was applied. Results indicated no significant difference in Nurse Time Damage between the Baseline and Replacement scenarios ($W=1853$, $p=0.611$). The mean values were highly comparable (Baseline: 8.87, Replacement: 9.22), suggesting that introducing an additional nurse in the Replacement scenario does not substantially affect the workload-related time damage for the high-performing nurse.

Patient Distribution Across Doctors: In the Baseline scenario (Scenario 1), some nurses show a preference for certain doctors, while in the Replacement scenario (Scenario 2), the distribution is slightly more even. Using a Monte Carlo x^2 test for each simulation run, 18 out of 60 runs in the Baseline scenario showed a statistically significant nurse preference for specific doctors ($p<0.05$). In the Replacement scenario, 12 out of 60 runs showed a significant preference.

The variance in the number of patients served per doctor per run was 21.44 for the Baseline scenario and 29.71 for the Replacement scenario. A paired t-test comparing these variances found no statistically significant difference ($p=0.938$).

Conclusion: Nurses occasionally favor certain doctors in both scenarios, but the evidence is not strong enough to indicate a systematic preference. Patient distribution across doctors is generally uneven in both Baseline and Replacement scenarios, with no significant difference in variance between them.

Conclusions

The Replacement scenario, which adds an extra nurse to handle high-difficulty tasks, substantially increases overall patient throughput and reduces patient delay. However, these operational gains come with the implicit cost of employing additional staff. While total patient damage time rises slightly, the main trade-off to consider is the financial and logistical burden of the extra nurse. Decision-makers should weigh the benefits of higher efficiency and reduced patient delays against the increased staffing costs, particularly in resource-constrained settings.

B.3. Baseline (CA trust model) vs. Training (CA trust model)

Objective

Evaluate the impact of assigning trainer nurses to accompany low-performing nurses within the CA Trust model. The focus is on whether this intervention improves overall system throughput and efficiency, while accounting for potential trade-offs in error rates, patient safety, and nurse performance outcomes.

Design

- Scenarios: Baseline vs. Training;
- Task selection policy: CA trust model applied in both scenarios;
- Metrics analyzed: Total patients served, total patient damage time, total patient delay, failed and successful tasks per nurse, nurse utility, nurse time damage, patient distribution across doctors.

Statistical Approach

All metrics were tested for normality using the Shapiro-Wilk test. Given that most metrics significantly deviated from normality, the non-parametric Wilcoxon rank-sum test was applied. For metrics with no variance in one group, the test was applied against the non-normal counterpart. Effect sizes, means, and p-values are reported. For patient distribution, Monte Carlo chi-square tests and variance comparisons via paired t-tests were conducted.

Key Results

Total Patients Served: Since both groups exhibited significant deviations from normality (Shapiro-Wilk $p < 2.2 \times 10^{-16}$), the non-parametric Wilcoxon rank-sum test was applied. Results showed that the Training scenario served significantly more patients $(W = 10850, p < 2.2 \times 10^{-16})$ compared to the baseline. The mean difference was approximately 6.93 patients per shift (Baseline: 22.18, Training: 29.12), indicating that assigning trainer nurses to accompany low-performing nurses substantially increases overall patient throughput.

Total Patient Damage Time: Since both groups deviated significantly from normality (Shapiro-Wilk $p < 2.2 \times 10^{-16}$ for Baseline, $p = 6.73 \times 10^{-5}$for Training), the non-parametric Wilcoxon rank-sum test was applied. Results showed that the Training scenario had significantly higher total patient damage time $(W = 7025, p < 2.2 \times 10^{-16})$ compared to the Baseline. The mean difference was approximately 83.9 seconds per shift (Baseline: 65.38 sec, Training: 149.28 sec), indicating that assigning trainer nurses increased the overall patient damage time.

Total Patient Delay: Since both groups exhibited significant deviations from normality (Shapiro-Wilk $p < 2.2 \times 10^{-16}$), the non-parametric Wilcoxon rank-sum test was applied. Results showed that the Training scenario had significantly lower total patient delay (W=83100, $p < 2.2 \times 10^{-16}$) compared to the Baseline. The mean difference was approximately 380.9 seconds per shift (Baseline: 7095.77 s, Training: 6714.88 s), indicating that implementing the training scenario reduces patient delays and potentially improves overall efficiency in the emergency department.

Failed Tasks by the Low-performing nurse: Since failures in the Baseline group exhibited significant deviations from normality (Shapiro-Wilk W=0.914, p=0.00045), while the Training group did not (W=0.967, p=0.102), the non-parametric Wilcoxon rank-sum test was applied. Results showed that the low-performing nurse committed significantly more failed tasks in the Training scenario ($W = 140, p < 2.2 \times 10^{-1}$) compared to the Baseline. The mean difference was approximately 4.28 failed tasks per shift (Baseline: 3.60, Training: 7.88). This outcome suggests that, although trainer nurses improve overall system throughput, low-performing nurses do not immediately reduce their error rate; instead, the improvement process requires gradual, long-term capability building.

Failed Tasks by the High-performing nurse: Since both Baseline and Training scenarios showed significant deviations from normality (Shapiro-Wilk p<0.001), the non-parametric Wilcoxon rank-sum test was applied. Results indicated no statistically significant difference in the number of failed tasks between the Baseline and Training scenarios (W=1675.5, p=0.5005). The mean number of failed tasks was similar across scenarios (Baseline: 1.92, Training: 2.08). These results suggest that the performance of the high-performing nurse remains stable between Baseline and Training, and the presence of trainers or the Training scenario itself does not significantly affect the number of failed tasks for this nurse.

Successful Tasks by the Low-performing nurse: Since the baseline group had identical values (all zeros), normality could not be assessed. The training group significantly deviated from normality (Shapiro-Wilk $W = 0.848, p = 2.72 \times 10^{-6}$), so the non-parametric Wilcoxon rank-sum test was applied. Results showed that the Training scenario achieved significantly more successful tasks ($W = 480, p = 9.86 \times 10^{-16}$) compared to the baseline. The mean difference was approximately 2.43 tasks per shift (Baseline: 0.00, Training: 2.43), indicating that training substantially improves the performance of low-performing nurses.

Successful Tasks by the High-performing nurse: Since both Baseline and Training scenarios exhibited significant deviations from normality (Shapiro-Wilk p<0.001 for both), the non-parametric Wilcoxon rank-sum test was applied. Results showed no statistically significant difference in the number of successful tasks between Baseline and Training (W=1958.5, p=0.403). The mean difference was minimal (Baseline: 16.67, Training: 16.72), indicating that training interventions to the High-performing nurse did not substantially affect task performance.

Nurse Utility by the Low-performing nurse: Since the Baseline group showed a significant deviation from normality (Shapiro-Wilk p=0.006) while the Training group did not

(p=0.941), the non-parametric Wilcoxon rank-sum test was applied. Results showed that the Training scenario had significantly lower Nurse Utility ($W = 551.5, p < 2.72 \times 10^{-11}$) compared to the Baseline. The mean difference was approximately -10.07 units (Baseline: -9.97, Training: -20.03), indicating that the Training scenario substantially reduces the utility of the low-performing nurse.

Nurse Utility by the High-performing nurse: Since both groups showed deviations from normality (Shapiro-Wilk p<0.05 for Baseline and Training), the non-parametric Wilcoxon rank-sum test was applied. Results indicated no significant difference in utility between the Training and Baseline scenarios (W=1588, p=0.868). The mean utility values were 50.28 for Baseline and 47.95 for Training. This suggests that assigning a trainer nurse did not substantially change the utility of the high-performing nurse in the simulation.

Nurse Time Damage by the Low-performing Nurse: Since the Baseline group exhibited significant deviation from normality (Shapiro-Wilk, p=0.0021), the non-parametric Wilcoxon rank-sum test was applied. Results showed that the Training scenario did not reduce Nurse Time Damage (W=3347.5, p=1) compared to the Baseline. The mean time damage actually increased from 56.48 in Baseline to 140.33 s in Training. This outcome aligns with findings on failed tasks: although trainer nurses improve overall system throughput, low-performing nurses do not immediately reduce their error rate; instead, the improvement process requires gradual, long-term capability building. These results highlight that training interventions may not immediately mitigate the inefficiencies caused by underperforming staff.

Nurse Time Damage by the High-performing Nurse: Since both groups deviated significantly from normality (Shapiro-Wilk p<0.005), the non-parametric Wilcoxon rank-sum test was applied. Results showed no significant difference in Nurse Time Damage between the Training scenario and the Baseline (W= 1811.5, p=0.5252). The mean Nurse Time Damage was 8.87 s in the Baseline and 8.92 s in the Training scenario, indicating that the good nurse’s performance remained stable. This aligns with our previous observation that while the trainer nurses can improve overall system throughput, individual low-performing nurses require gradual capability building; high-performing nurses maintain consistent efficiency regardless of scenario.

Patient Distribution Across Doctors: In the Baseline scenario, some nurses show a preference for certain doctors, while in the Training scenario, the distribution is slightly more even. Using a Monte Carlo chi-square test for each simulation run, 18 out of 60 runs in the Baseline scenario showed a statistically significant nurse preference for specific doctors

(p<0.05). In the Training scenario, 15 out of 60 runs showed a significant preference.

The variance in the number of patients served per doctor per run was 21.44 for the Baseline scenario and 26.29 for the Training scenario. A paired t-test comparing these variances found no statistically significant difference (p=0.838).

Conclusion: Nurses occasionally favor certain doctors in both scenarios, but the evidence is not strong enough to indicate a systematic preference. Patient distribution across doctors is generally uneven in both Baseline and Training scenarios, with no significant difference in variance between them.

Conclusions

The Training scenario substantially increases the number of patients served and reduces total patient delays demonstrating a clear improvement in system throughput under the use of CA Trust model. However, this comes at the cost of higher patient damage time and increased failed tasks by the low-performing nurse, highlighting that the error rate does not improve immediately. Instead, capability building for underperforming staff appears to be gradual and long-term in nature. The presence of trainer nurses stabilizes outcomes for high-performing nurses, whose performance and utility remain largely unaffected across scenarios.

From a managerial perspective, the Training scenario illustrates a critical trade-off: while system-level efficiency improves, short-term patient safety risks may increase due to persistent errors by low-performing nurses. Decision-makers should recognize that training interventions deliver operational gains but require sustained investment in long-term skill development to mitigate the risks associated with low-performing staff.

B.4. Replacement (CA trust model) vs. Training (CA trust model)

Objective

Compare two intervention strategies for handling low-performing nurses: immediate replacement vs skill development under training. Specifically, the study investigates how these interventions affect patient throughput, patient safety, nurse performance, and overall system efficiency.

Design

- Scenarios: Baseline vs. Training;
- Task selection policy: CA trust model applied in both scenarios;
- Metrics analyzed: Total patients served, total patient damage time, total patient delay, failed and successful tasks per nurse, nurse utility, nurse time damage, patient distribution across doctors.

Statistical Approach

All metrics were tested for normality using the Shapiro-Wilk test. Given that most metrics significantly deviated from normality, the non-parametric Wilcoxon rank-sum test was applied. For metrics with no variance in one group, the test was applied against the non-normal counterpart. Effect sizes, means, and p-values are reported. For patient distribution, Monte Carlo chi-square tests and variance comparisons via paired t-tests were conducted.

Key Results

Total Patients Served: Since both groups deviated significantly from normality (Shapiro-Wilk test, $p < 2.2 \times 10^{-1}$), the non-parametric Wilcoxon rank-sum test was applied. Results showed that the Replacement scenario served significantly more patients (W=104114, $p < 2.2 \times 10^{-16}$) compared to the Training scenario. The mean difference was approximately 12.47 patients per shift (Replacement: 41.58, Training: 29.12), indicating that immediate replacement of low-performing nurses results in higher patient throughput compared to training.

Total Patient Damage Time: Since both groups exhibited significant deviations from normality (Shapiro-Wilk test, Replacement: $= 0.982, p = 0.00016$; $Training: W = 0.976, p = 6.73 \times 10^{-5}$), the non-parametric Wilcoxon rank-sum test was applied. Results showed that the Replacement scenario had significantly lower total patient damage time compared to the Training scenario ($W = 19695, p < 2.2 \times 10^{-16}$). The mean difference was approximately 53.3 seconds per shift (Replacement: 95.97 s, Training: 149.28 s), indicating that replacing low-performing nurses with more capable ones substantially reduces the overall damage exposure time of patients in the emergency department.

Total Patient Delay: Since both groups deviated significantly from normality (Shapiro-Wilk test, $p < 2.2 \times 10^{-16}$), the non-parametric Wilcoxon rank-sum test was applied. Results showed that the Replacement scenario had significantly lower patient delay (W=4440, $p < 2.2 \times 10^{-16}$) compared to the Training scenario. The mean difference was approximately 430.77 seconds per shift (Replacement: 6284.12, Training: 6714.88), indicating that immediate replacement of low-performing nurses reduces total patient delay more effectively than training.

Failed Tasks by the Low-performing nurse: Since the Replacement group deviated significantly from normality (Shapiro-Wilk test, $W = 0.908, p = 2.66 \times 10^{-4}$), while the Training group was approximately normal (W=0.967,p=0.102), the non-parametric Wilcoxon rank-sum test was applied. Results showed that the low-performing nurse had significantly fewer failed tasks in the Replacement scenario compared to the Training scenario (W=156.5, $p < 2.2 \times 10^{-16}$). The mean

difference was approximately 3.88 failed tasks per shift (Replacement: 4.00, Training: 7.88), indicating that immediate replacement of low-performing nurses reduces task failures compared to training.

Failed Tasks by the High-performing nurse: Since both groups deviated significantly from normality (Shapiro-Wilk test, Replacement: W=0.910, p=0.0003; Training: W=0.860, $p = 6.3 \times 10^{-6}$), the non-parametric Wilcoxon rank-sum test was applied. Results showed no statistically significant difference between Replacement and Training (W=1727.5, p=0.695). The mean number of failed tasks was similar across conditions (Replacement: 1.92, Training: 2.08).

Successful Tasks by the Low-performing nurse: Since the Replacement group consisted of identical values (all zero) and the Training group deviated significantly from normality (Shapiro-Wilk test, $W = 0.848, p = 2.72 \times 10^{-6}$), the non-parametric Wilcoxon rank-sum test was applied. Results showed that the Training scenario produced significantly more successful tasks ($W = 480, p < 9.86 \times 10^{-16}$) compared to the Replacement scenario. The mean difference was approximately 2.43 successful tasks per shift (Replacement: 0.00, Training: 2.43), indicating that training substantially improved the performance of low-performing nurses compared to immediate replacement.

Successful Tasks by the High-performing nurse: Since both groups deviated significantly from normality (Shapiro-Wilk test, Replacement: $W = 0.813, p = 2.97 \times 10^{-7}$; Training: $W = 0.747, p = 7.98 \times 10^{-9}$), the non-parametric Wilcoxon rank-sum test was applied. Results showed that the Replacement scenario yielded significantly more successful tasks compared to the Training scenario (W=2269, p=0.013). The mean difference was approximately 1.20 tasks per shift (Replacement: 17.92, Training: 16.72). The results suggest that immediate replacement of the low-performing nurse leads to a modest but statistically significant improvement in the performance of the high-performing nurse, as measured by the number of successful tasks.

Nurse Utility by the Low-performing nurse: Since the Replacement group deviated significantly from normality (Shapiro-Wilk test, W=0.922, p=0.0009), while the Training group did not (W=0.991, p=0.94), the non-parametric Wilcoxon rank-sum test was applied. Results showed that the Replacement scenario yielded significantly higher utility for the low-performing nurse compared to the Training scenario ($W = 3038, p = 7.85 \times 10^{-11}$). The mean difference was approximately 9.57 utility points (Replacement: -10.47, Training: -20.03).

Nurse Utility by the High-performing nurse: Since both groups significantly deviated from normality (Shapiro-Wilk

test, Replacement: W=0.951, p=0.018; Training: W=0.958, p=0.036), the non-parametric Wilcoxon rank-sum test was applied. Results showed no significant improvement of nurse utility in the Training scenario compared to Replacement (W=1189, p=0.999), with mean utility being higher under Replacement (56.45) than Training (47.95).

Nurse Time Damage by the Low-performing nurse: Since the Replacement group significantly deviated from normality (Shapiro-Wilk test, Replacement: W= 0.938, p=0.0045) while the Training group was approximately normal (W=0.974, p=0.230), the non-parametric Wilcoxon rank-sum test was applied. Results showed a significant increase in nurse time damage in the Training scenario compared to Replacement ($W = 3384, p < 2.2 \times 10^{-16}$), with mean time damage being substantially higher under Training (140.33) than Replacement (58.07).

Nurse Time Damage by the High-performing nurse: Since both groups significantly deviated from normality (Shapiro-Wilk test, Replacement: W=0.946, p=0.011; Training: W=0.937, p=0.004), the non-parametric Wilcoxon rank-sum test was applied. Results showed no significant difference in nurse time damage between the Training and Replacement scenarios (W=1750.5, p=0.604), with mean time damage being slightly lower under Training (8.92) than Replacement (9.22).

Patient Distribution Across Doctors: In the Replacement scenario, some nurses show a preference for certain doctors, while in the Training scenario, the distribution is slightly more even. Using a Monte Carlo chi-square test for each simulation run, 12 out of 60 runs in the Replacement scenario showed a statistically significant nurse preference for specific doctors (p<0.05). In the Training scenario, 15 out of 60 runs showed a significant preference.

The variance in the number of patients served per doctor per run was 29.71 for the Replacement scenario and 26.29 for the Training scenario. A paired t-test comparing these variances found no statistically significant difference (p=0.274).

Nurses occasionally favor certain doctors in both scenarios, but the evidence is not strong enough to indicate a systematic preference. Patient distribution across doctors is generally uneven in both Replacement and Training scenarios, with no significant difference in variance between them.

Conclusions

Replacement of low-performing nurses substantially improves patient throughput, reduces patient damage time, and lowers total patient delay compared to training. Training, however, enables low-performing nurses to achieve more successful tasks. The performance of high-performing nurses improves slightly under Replacement, while overall nurse utility favors Replacement. Patient distribution across doctors remains uneven but comparable across scenarios.

The Replacement scenario provides clear operational benefits, including faster patient service and reduced cumulative damage and delay. Training improves the skills of low-performing nurses, increasing their successful tasks but at the cost of higher patient delay and damage. Decision-makers must weigh the trade-off between immediate efficiency gains from Replacement versus long-term skill development through Training, especially when staffing resources are limited.
